# Guide Picker is a comprehensive design tool for visualizing and selecting guides for CRISPR experiments

**DOI:** 10.1186/s12859-017-1581-4

**Published:** 2017-03-14

**Authors:** Soren H. Hough, Kris Kancleris, Leigh Brody, Neil Humphryes-Kirilov, Joseph Wolanski, Keith Dunaway, Ayokunmi Ajetunmobi, Victor Dillard

**Affiliations:** Desktop Genetics, Ltd., 28 Hanbury Street, London, E1 6QR UK

**Keywords:** CRISPR, sgRNA, RNA guide design software, Cas9, Genome editing, Functional genomics

## Abstract

**Background:**

Guide Picker (https://www.deskgen.com/guide-picker/) serves as a meta tool for designing CRISPR experiments by presenting ten different guide RNA scoring functions in one simple graphical interface. It allows investigators to simultaneously visualize and sort through every guide targeting the protein-coding regions of any mouse or human gene.

**Results:**

Utilizing a multidimensional graphical display featuring two plots and four axes, Guide Picker can analyze all guides while filtering based on four different criteria at a time. Guide Picker further facilitates the CRISPR design process by using pre-computed scores for all guides, thereby offering rapid guide RNA generation and selection.

**Conclusions:**

The ease-of-use of Guide Picker complements CRISPR itself, matching a powerful and modular biological system with a flexible online web tool that can be used in a variety of genome editing experimental contexts.

## Background

CRISPR (clustered regularly interspaced short palindromic repeats) allows researchers to introduce site-specific mutations in a variety of organisms [1, 2]. SpCas9 (an RNA-guided nuclease found in *Streptococcus pyogenes*) is directed to target sites in the genome by a chimeric single guide RNA (sgRNA) [1]. The sgRNA forms a complex with Cas9 and binds to genomic DNA according to a 20 bp protospacer sequence. The complex then induces a double-stranded break (DSB) three nucleotides upstream of the protospacer adjacent motif (PAM). The cell usually repairs the DSB through the endogenous non-homologous end joining (NHEJ) pathway which often produces insertion/deletion (indel) and potentially deleterious frameshift mutations [3]. Customizing the 20 bp protospacer elements of the sgRNAs to target within and across different genes allows researchers to multiplex functional genomics experiments.

The PAM is essential for Cas9 binding. SpCas9 primarily recognizes NGG PAMs [4]. Other PAMs, such as NAG, are referred to as non-canonical and have much lower rates of cleavage [4]. However, although NAG is not as strong as NGG, SpCas9 may still cleave near NAG PAMs. Therefore, NAG PAMs are relevant when searching for off-target hits but are not desirable when designing highly active guides [4]. SpCas9 also has tolerance for mismatches in the 20 bp protospacer element and can still induce DSBs despite a lack of full complementarity [5]. In concert, variable PAM sequences and mismatch tolerance can lead to off-target edits (often via NHEJ) in unintended regions across the genome. These characteristics should be considered when predicting and analyzing off-target activity.

To ensure target specificity and guide activity, researchers depend on intelligent guide RNA design tools to predict guide RNA behavior [6]. Several algorithms have already been released which use guide RNA sequences as predictors of both on- [7, 8] and off-target [4, 8] activity based on sequence composition. Additional algorithms focus on GC content [9], homopolymers [10] and other features. Existing online web tools frequently offer one or combine a few design considerations, but rarely aggregate all of these parameters in one place. This forces investigators to spend time comparing across multiple websites in order to guarantee optimal guide RNA design.

**Fig. 1:**
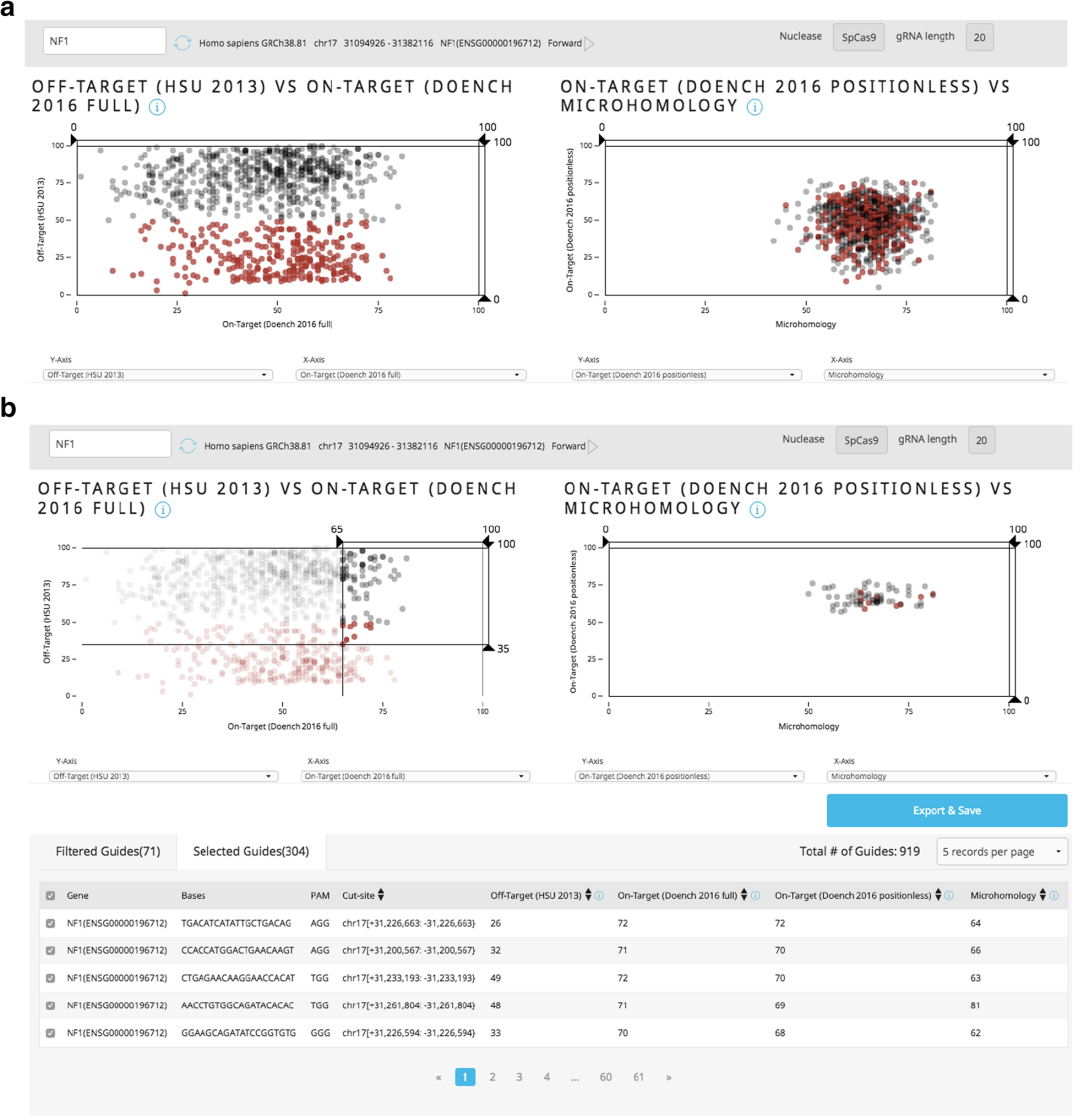
Visualizing and Filtering Guides. **a** Guide sequences can be selected as a group or individually based on user preference. Selecting the guide RNA sequence marker from the left-hand plot will highlight the same guides on the right-hand plot in red. **b** Users can filter guide RNA sequences in the left-hand plot to minimize the population in the right-hand plot. This can be done by clicking and dragging the filtering tool on the x- and/or y-axis. Unselected guides will fade out on the left-hand side and disappear altogether on the right. Selected guides will be stored in the “Selected Guides” table for further analysis

To address these problems, we developed Guide Picker. Guide Picker is a cloud-based tool that allows the user to visualize guide RNA designs plotted according to ten scoring functions using one simple graphical interface. Guide Picker can compare on- and off-target scores, as well as other parameters, for every guide RNA targeting the protein-coding transcripts in a given mouse or human gene. Filtering and selecting guides according to different scores in one interface alleviates the labor involved in testing designs across disparate guide RNA design tools (Fig. 1). Once the user has generated suitable designs, the list of guide RNAs can be saved and passed on for synthesis and experimental application.

Guide Picker is also unique because it is the only online resource that allows guide design around all protein-coding transcripts of a gene. Transcripts are identified using Ensembl database annotations indicating known coding DNA sequences. Some design tools limit guide design to a 250 nucleotide input sequence while others only generate guides for a single transcript. By using all transcripts for a given gene, Guide Picker can offer more guide design options and help the user target as many transcript variants as possible to ensure gene knockout.

Guide sequences are determined by performing an exhaustive search throughout all protein-coding regions of the mouse or human genome based solely on available NGG SpCas9 PAM sites. This is accomplished using in-house Python scripts which, along with the scores, are contained in a Python wrapper to facilitate automation. This loading process occurs on a cloud-based web server and not on the user’s computer.

In addition to pre-loading guide sequences, Guide Picker further speeds up the CRISPR design process by pre-computing all scores for every guide RNA targeting coding genomic regions in the mouse and human reference genomes. For any given scoring function and gene, rendering all available guides takes fewer than five seconds (even for large genes with ~3000 guides, such as *MUC4*). Guide Picker displays all of these guides in an easily manageable graphical format that can be adjusted to improve visual accessibility.

## Implementation

### Materials and methods

The Guide Picker user interface (UI) is built on custom in-house components developed using open source libraries Vue.js (https://github.com/vuejs/vue), a JavaScript framework, and a data visualization library, D3.js (https://github.com/d3/d3). Interfacing with the open source community assured maintainability and compatibility of the Guide Picker tool with most modern web browsers. Using a powerful D3.js library enabled fast prototyping and development of the graphic component, as well as access to its advanced data visualization algorithms such as quadtree (https://github.com/d3/d3-quadtree) which is used in the “Force Layout” mode to detect collisions between data points (Fig. 3).

All Guide Picker scoring functions are based on previously published studies. Further, Guide Picker is free and accessible to the academic community on http://deskgen.com without restriction following sign-up. There is no paid version of Guide Picker. Moreover, the source code for Guide Picker at the time of publication is available in a GitHub repository at https://github.com/DeskGen/open-guide-picker under an open source MIT license. This includes access to the pre-computed guide score database associated with the tool.

Guide Picker uses the reference genomes for *Mus musculus* (GRCm38) and *Homo sapiens* (GRCh38) provided by Ensembl. This is because the scoring functions provided in the tool were developed with datasets from mammalian models and depended on standard U6 delivery plasmid systems. Therefore, all algorithms displayed by Guide Picker are constructed to make predictions within that context [6]. Similarly, Guide Picker only uses SpCas9 guide RNA design rules. We made this decision because all guide RNA scoring algorithms to date were written to accommodate this nuclease and not its orthologs (e.g. NmCas9) which vary in PAM recognition, specificity and more.

### User interface

The number of relationships between guide RNA data points can be explained by the formula *n* ^*p*^ (where *n* is a number of guides and *p* is a number of properties). The multidimensionality of guide RNA data yields an unwieldy number of variables for an investigator to navigate during the guide RNA design process. It also presents a challenge for creating a simple and efficient web tool UI. Guide Picker arose as a solution to this problem.

A previously trialed UI (https://www.deskgen.com/guidebook/advanced.html), where the user selected guides by manually navigating inside genes with a sequence browser, turned out to be an impractical, labor-intensive solution. In parallel, an internally used algorithm-assisted UI which presented the user with five top scoring guides for a given gene limited user engagement and control. It also stymied the user’s ability to cross-validate guides across multiple parameter thresholds.

To achieve maximum clarity and usability for this tool, a deliberate choice was made to adopt the minimalist design paradigm by reducing the subject to its principal components. This philosophy yielded a simple yet effective scatter plot graphic. The scatter plot allowed users to visualize and compare thousands of guides at once, thereby providing an intuitive UI for selecting guides that met specific scoring thresholds. It also offered more control over design than the algorithm-assisted UI.

However, any gains in clarity over previous UI iterations and peer web tools were outweighed by a reduction in data depth. The two-dimensional x/y view did not present a comprehensive method for selecting optimal guides. Also, a number of relationships between data points *C(n, k)* (where *n* is the number of properties and *k* is the number of axes) produced an unworkable UI. To compensate for this loss of dimensionality, we drew two scatter plots side-by-side, each displaying different properties for the same set of guides. The axis values of these two plots were user-selected guide design parameters. This improved readability while maintaining a robust level of customizable dimensionality.

We also chose to round score values to the nearest integer. Although floating numbers would provide a more continuous distribution of values, they are harder for users to read and compare. A decimal point is also likely to be statistically insignificant for choosing an optimal guide. Therefore, we decided to round score values as is consistent with other guide RNA design web tools [4, 8].

Due to the volume of guide RNA data and our decision to round score values to the nearest integer, highly dense overlapping regions became common in the scatterplots. In order to explore these dense regions more easily, we implemented a “Force Layout” view and “Fisheye” lensing (advanced Guide Picker features) to allow users to visualize overlapping guides or guides in close proximity to one another (Fig. 3). In concert, displaying all guides for a gene side-by-side across two plots according to four variable guide RNA scores offers unprecedented ease and control over guide design.

## Results

### How to use Guide Picker

#### Input

**Fig. 2:**
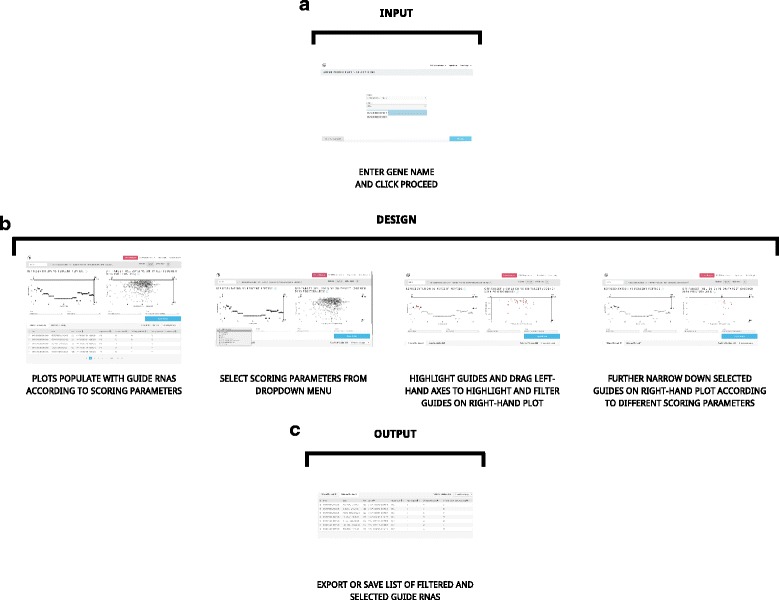
Guide Picker Workflow. **a** First, an Ensembl gene name is provided from either the mouse or human reference genome. **b** Then, the guide RNAs populate the left- and right-hand plots, organized on axes according to the scores selected in the corresponding dropdown menus. Guides are filtered and selected based on these parameters. **c** Finally, a list of guide sequences are output and can be saved or sent to an oligo synthesis provider

The user first selects the genome of interest from the “Genome” drop-down menu: either *Homo sapiens* (GRCh38) or *Mus musculus* (GRCm38). The user then inputs the gene name into the “Gene” entry field. The system will search for the Ensembl gene name and list it in a drop-down menu. Once the gene is selected, the user clicks “Proceed” to be taken to the main Guide Picker interface (Fig. 2a).

#### Design

**Fig. 3:**
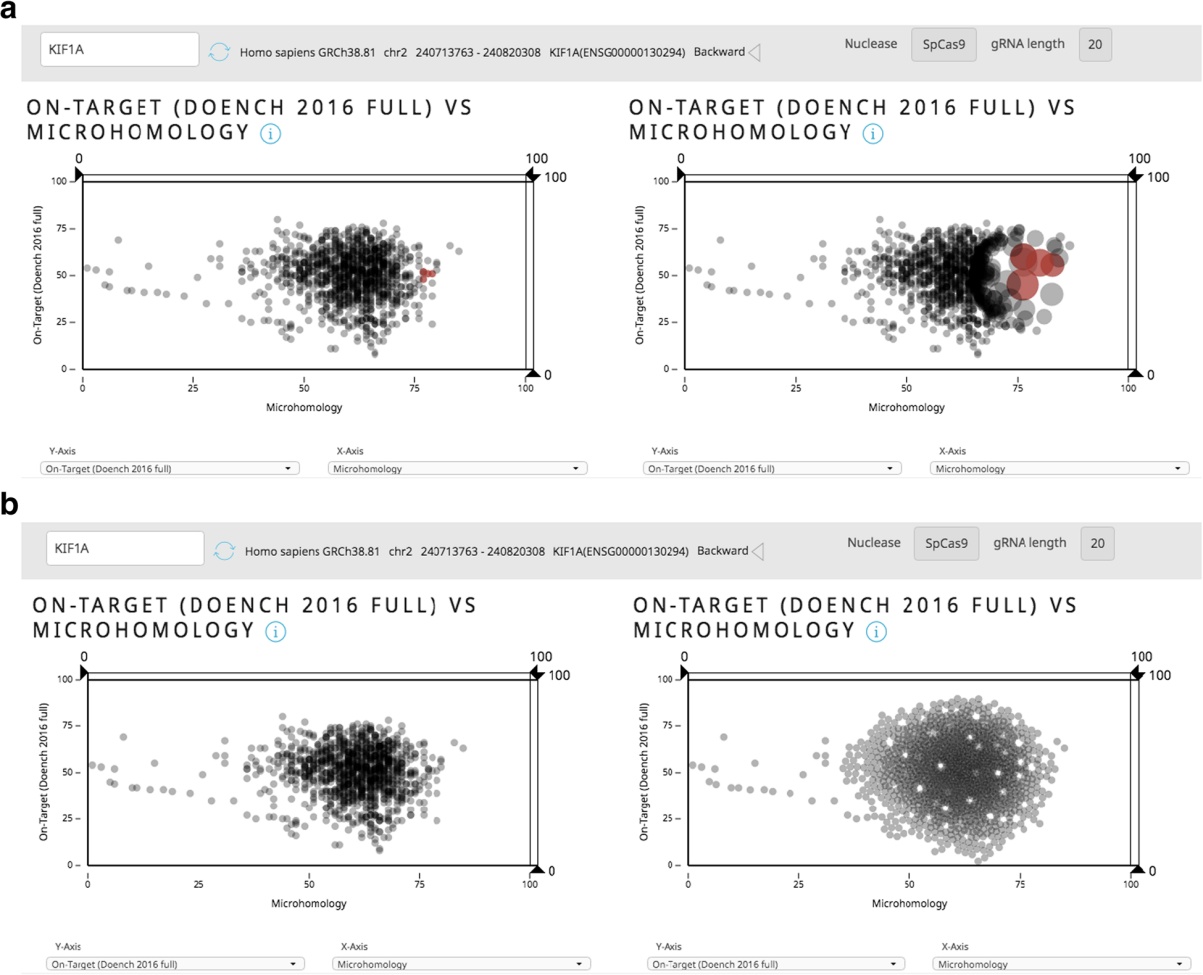
Advanced Guide Picker Tools. By right-clicking on the plots in Guide Picker, the user can access advanced visualization options to more easily view and select guides. **a** The “Fisheye” tool allows users to repurpose their mouse as a magnifying glass, giving them the ability to more easily select specific guides of interest. **b** The “Force Layout” tool allows users to spread the overlapping guide RNA markers apart to see and select them more clearly

In the main interface, side-by-side plots populate with black data points. Each point represents an individual guide RNA targeting the coding regions of the selected gene using NGG PAM sites. Guides can be selected by clicking data points directly or by Shift-clicking and dragging the crosshair. Selected guides will change color to red. Right-clicking will open a menu to select either Fisheye or Force Layout view (Fig. 3). Axes can be dragged to filter guides based on score thresholds. Drop-down menus below each plot can be used to reassign x- and y-axes to various scoring functions (Fig. 2b).

##### Scoring parameters

The scatterplots on Guide Picker depict every guide RNA available to target within the coding regions of the specified gene. Guide RNAs are organized by the x and y plot scoring variables determined by drop-downs underneath each scatterplot. Most scoring functions are continuous (from 0 to 100), but some are either stepped (0,10 … 90,100) or binary (0 or 100, true or false).

##### Percent peptide score

**Fig. 4:**
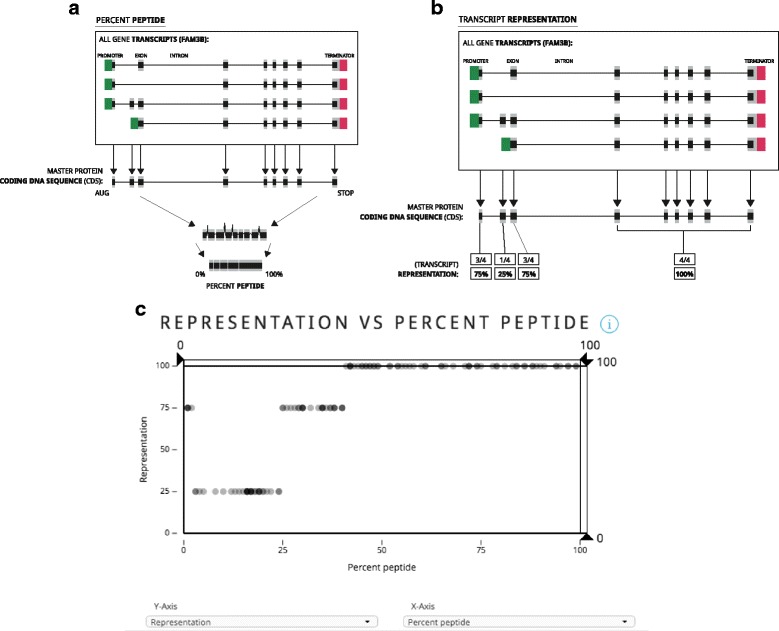
Percent Peptide and Transcript Representation. **a** The Guide Picker tool uses percent peptide as an indicator of location within the master coding DNA sequence (MCDS) of a gene. A score lower than 50% means the guide RNA targets closer to the 5′ end of the MCDS, while a score greater than 50% means the guide RNA targets toward the 3′ end of the gene. **b** Transcript representation refers to the number of gene transcripts this guide RNA can target with the same gRNA sequence. **c** Plotting percent peptide and transcript representation together reveals consensus exons throughout the MCDS of a given gene. In this example, most consensus exons appear toward the 3′ end of the gene. Data shown for the human (GRCh38) *FAM3B* gene. Introns are not drawn to scale

The percent peptide score (PPS) refers to the guide position within the protein-coding portion of the entire gene. In Guide Picker, protein-coding exons for each transcript are concatenated together from the ATG/AUG codon to the STOP codon and multiple transcripts are overlaid to produce one theoretical master coding DNA sequence (MCDS) per gene (Fig. 4a). The base pair values for this sequence are normalized from 0 to 100, 5′–3′ to provide percentage progression through the MCDS. Guides with a PPS of <50% target toward the 5′ half of the MCDS, while guides with a >50% PPS target the 3′ half.

##### Transcript representation

Representation refers to the proportion of the gene’s protein-coding transcripts a given guide RNA design can target. The stepped axis levels represent the percentage of targeted transcripts versus the total transcripts for that gene. This value is useful for designing guides against highly represented (consensus) exons. A graphical representation of how Transcript Representation is computed can be found in Fig. 4b. Plotting PPS against Transcript Representation in Guide Picker can be useful to determine the location of highly conserved transcripts in the context of the MCDS (Fig. 4c).

##### Specificity score (Hsu 2013)

The Hsu 2013 score predicts the specificity of the guide RNA. Off-target sites are evaluated based on genomic similarity to the guide RNA sequence. This evaluation takes into account mismatch number, position and density [4]. It is important to note that while Hsu 2013 evaluates mismatch position and nucleotide number, it does not consider nucleotide identity (ATGC) [8].

Hsu 2013 considers both canonical NGG and non-canonical NAG PAM sites for SpCas9 [4]. This information is accumulated into a continuous score from 0 to 100. A higher score indicates the guide is less likely to direct SpCas9 to cut at unintended (off-target) sites in the genome. A score of over 50 means the guide has no exact matches elsewhere in the genome, and a score of 100 represents maximum specificity.

##### On-target activity score (Doench 2014)

The Doench 2014 on-target activity score predicts the ability of the guide RNA to knock out the target gene [7]. This score was developed based on a large-scale CRISPR experiment using 1841 guide RNAs saturating nine genes [7]. The group investigated position-based nucleotide composition for guide RNAs with high versus low activity and constructed a scoring algorithm according to desirable/undesirable sequence traits [7]. A score of 100 represents the highest predicted activity based on nucleotide sequence.

##### On-target activity (Doench 2016 full and positionless)

Like the Doench 2014 score, the Doench 2016 on-target activity score also predicts the ability of the guide RNA to knock out the target gene [8]. This score is an improvement on Doench 2014 because it ranks data from multiple large-scale CRISPR experiments and combines their information to build a new algorithm with a more generalizable model [8]. Once again, the group investigated the nucleotide composition of the guide RNAs and compared this data to activity [8]. A score of 100 represents the highest predicted guide RNA activity based on nucleotide sequence. Guide Picker uses the latest version of the Doench algorithm available through the Azimuth GitHub (https://github.com/MicrosoftResearch/Azimuth).

Doench 2016 comes in two forms: Full and Positionless. The Doench 2016 Full score is adjusted based on the target location in the coding DNA sequence while the Doench 2016 Positionless score does not. This adjustment is based on the percent peptide score (PPS) which represents the progression through the CDS of that gene.

The reason to consider accounting for position in the CDS is that some studies have suggested that targeting in the 3′ end of the gene is less likely to lead to gene knockout [8], possibly due to nonsense-mediated decay [11]. Therefore, Doench 2016 Full scores tend to be lower near the 3′ end of the gene. Conversely, Positionless does not penalize for targeting in the last third of the CDS.

##### GC content

Extreme GC content (low or high) can lead to poor or depleted guide RNA activity. The percentage of GC content refers specifically to the guide RNA protospacer element (not including the PAM). A recent study concluded that a range of 30–70% GC content yields optimal guide RNA activity [9]. GC content is implemented in Guide Picker as a continuous score of 0–100%.

##### Homopolymer score

Four or more consecutive repeated nucleotides (homopolymers) in the guide RNA sequence have been shown to be detrimental to guide RNA activity [10]. The homopolymer score in Guide Picker (“No Homopolymer”) yields a binary true/false output. A score of 100 means the guide RNA does not contain a consecutive 4+ nucleotide homopolymer (desirable) and a score of zero means it does contain a 4+ nucleotide homopolymer (undesirable).

##### Uracil triplets (UUU) score

The presence of a TTT DNA sequence (UUU in the RNA product) is detrimental to guide RNA activity because it is a terminator sequence for RNA Pol III transcription [12]. Like the homopolymer score, the uracil triplet score in Guide Picker (“No UUU”) is binary. A score of 100 means the guide RNA does not contain any TTT sequences (desirable) and a score of zero means it does contain at least one TTT sequence (undesirable).

##### Microhomology score

The microhomology score predicts the likelihood of creating an out-of-frame mutation via NHEJ-mediated repair [13]. Regions of microhomology close to the cut site can facilitate indel formation, The higher the score, the more likely the guide RNA is to produce a frameshift-causing indel (desirable for knockout experiments).

#### Output

Once the guide RNA designs have been filtered and selected, the Guide Picker-generated list can be saved for later use. The “Export & Save” button will download a .CSV report on all selected guides and store guide information in the My Projects tool (https://www.deskgen.com/my-projects/) at DESKGEN.com. The list is also text-selectable and can be copy-pasted by the user into a separate document (Fig. 2c).

#### Score comparisons

**Fig. 5:**
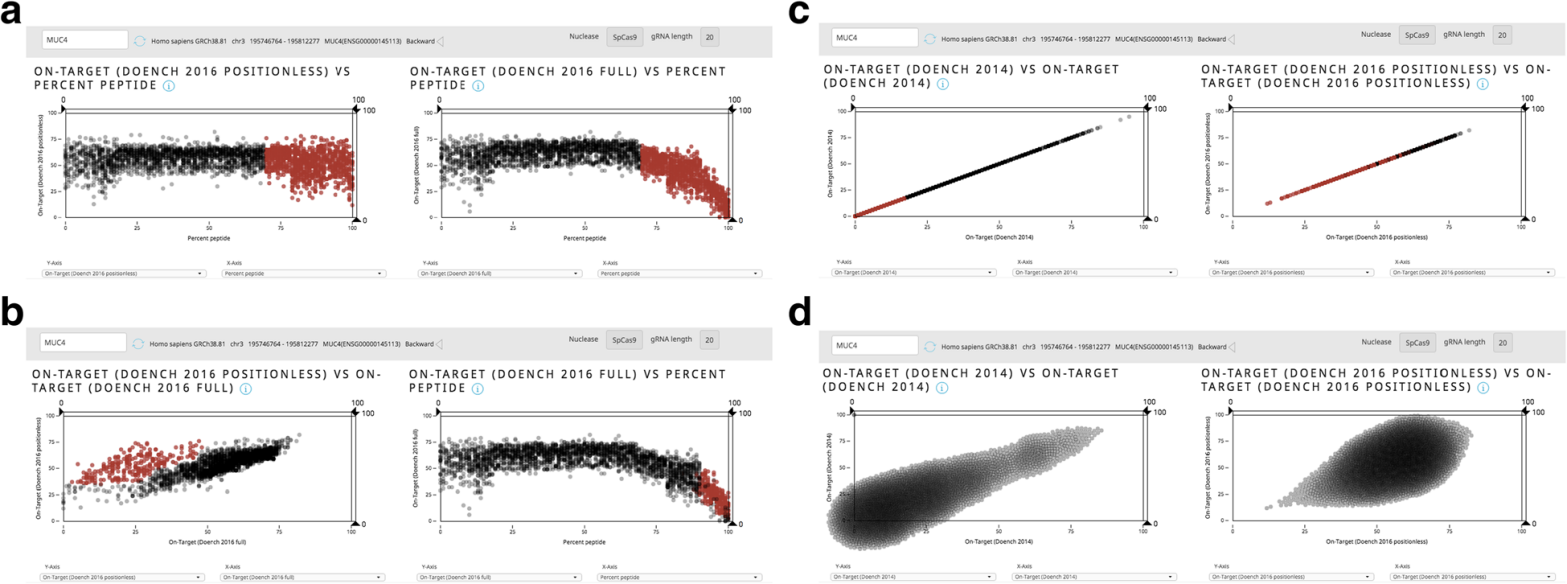
Score Comparisons. Each screenshots depict side-by-side plots containing all guides targeting the MCDS of *MUC4*. **a**–**b** Plotting Doench 2016 Full and Positionless against percent peptide reveals a scoring bias against guides targeting the 3′ end of the MCDS in Doench 2016 Full. **c** Guide Picker can also be used to illustrate score distribution patterns for different scores. In these plots, Doench 2016 Positionless guide scores cluster toward the <50 end of the plot while Full guide scores tend to cluster around 50. **d ** This is demonstrated even more clearly using Force Layout mode

Guide Picker can be used to compare scoring functions across all guides targeting a single gene’s MCDS. This can illuminate trends and biases in scoring functions. The visualizations in Fig. 5 were performed using the human Mucin 4 (*MUC4*) gene as an example. Due to the size of *MUC4*, it has more guides than many other genes and therefore demonstrates these parameter relationships more clearly.

The two plots in Fig. 5a illustrate how Guide Picker can be used to visualize key features of well-known scoring functions. The plot on the left compares the Doench 2016 Positionless on-target score with PPS, representing the location within the MCDS. Of note, there is no clear bias from Doench 2016 Positionless against guide scores at any position.

The plot on the right of Fig. 5a shows the same guides, but the y-axis displays Doench 2016 Full instead of Positionless. The downward trend toward a PPS of 100, or the 3′ end of the MCDS, demonstrates the bias of the Doench 2016 Full score. Doench 2016 Full operates under the assumption that guides targeting the 3′ end of the gene are less likely to induce gene knockout.

The difference between Full and Positionless is also made apparent in Fig. 5b. Correlating Doench 2016 Full and Positionless shows strong co-localization for many of the guide scores. However, some guides score much higher with Doench 2016 Positionless than they do with Full. Based on the relationships shown in Fig. 5a, it follows that the cluster of non-correlated guides would be located toward the 3′ end of the gene. We demonstrated this point by highlighting the non-correlated guide cluster in red in the plot on the left, which in turn showed the same guides with much lower Full scores toward 100% PPS on the right-hand plot.

Comparing Doench 2016 Full and Positionless in this way helps the user elucidate the difference between these two scores. Practically speaking, some investigators may want to avoid targeting the 3′ end of the gene and therefore will want to use Doench 2016 Full. Others may not rely on generalizations about the 5′ or 3′ end of the gene and instead will want to target 3′ proximal functional domains where appropriate in order to ensure gene knockout [14].

Guide Picker can also be used to demonstrate distributions for various scores by plotting parameters against themselves. In Fig. 5c, we compared Doench 2014 (left) and Doench 2016 Positionless (right) because they both avoid taking target location within the MCDS into account. Here we show that Doench 2014 tends to give guides a much lower score than Doench 2016 Positionless on average. We do so by highlighting about 1400 guides on the Doench 2014 plot which have lower Doench 2014 scores. With the Doench 2016 Positionless score, the distribution trends toward the middle of the plot. This is accentuated using the Force Layout feature (Fig. 5d).

## Discussion

Guide Picker can be used to design both gene knockout and tiling experiments using SpCas9. For gene knockout, the scoring parameters can be set to maximize guide efficiency. Ideally, this means selecting guides with a high Doench 2016 score, a high Hsu 2013 score, no homopolymers or uracil triplets, high transcript representation and a high microhomology score. Location in the MCDS (as indicated by the percent peptide score) will vary depending on the experiment.

**Fig. 6:**
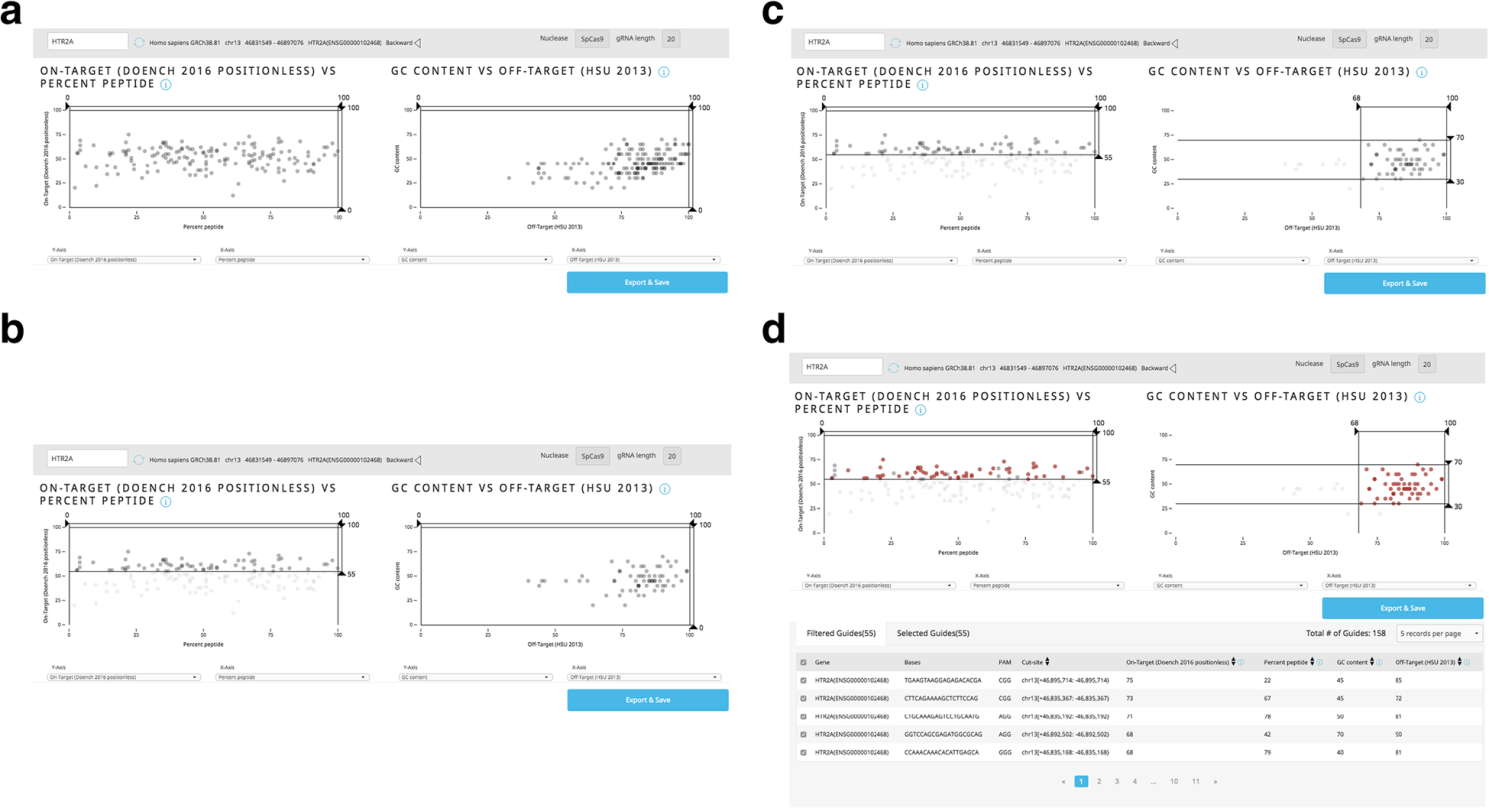
Designing a Tiling Experiment with Guide Picker. **a** First, guides are plotted along the length of the MCDS using percent peptide on the x-axis. The y-axis is set to Doench 2016 Positionless. **b** Filtering guides to a strict score threshold (e.g. <55) offers a panel of guides that still target along the full length of the MCDS. **c** The right-hand plot can then be set and filtered according to other guide parameters, such as GC (30–70%) content and Hsu 2013 (<68) off-target scoring. **d** The guides in this range can then be highlighted to verify that this select group of guides still target along the full length of the MCDS in the left-hand plot

Guide Picker can also be used to interrogate specific regions of a gene in what is known as a CRISPR tiling experiment. In principle, this involves systematically designing guides that target along the MCDS to determine regional essentiality in protein function [15]. This can be done efficiently with the Guide Picker interface (Fig. 6).

First, guides are displayed in a PPS versus Doench 2016 Positionless on-target score plot. This is displayed in Fig. 6a using the human serotonin receptor 2A gene, *HTR2A*, as an example. We use Positionless in this example because we do not want to introduce scoring bias against guides targeting the 3′ end of the gene; 3′ regions may still be functional or essential. The guides can then be filtered according to a Doench 2016 Positionless threshold (e.g. 50) (Fig. 6b). The “Filtered Guides” list is now restricted to designs that target along the whole MCDS and that have a higher likelihood of on-target activity.

All guides above this threshold are then brought over to the right-hand plot and further narrowed down according to, for example, a relatively high Hsu 2013 off-target score (68, which is >50 and means they have no exact matches elsewhere in the genome) and a GC content range of 30–70% [9] (Fig. 6c). The final guide selection can be highlighted on the right to verify that they still target along the full length of the MCDS (as evidenced by a broad range of PPS values on the left-hand plot) (Fig. 6d).

In this example, the user now has 55 guides with convincing GC content, on-target scores and off-target scores that will direct SpCas9 across the entirety of the MCDS of *HTR2A*. These guide sequences can then be checked in the Knockout or Knockin tools (https://www.deskgen.com/guidebook/) or a comparable genome browser to determine their exact location in the transcript(s) or MCDS. From there, the user can execute the experiment using a small-scale library generated with the list from Guide Picker to elucidate the essentiality of each protein-coding region.

## Conclusions

Guide Picker is the newest addition to the DESKGEN [16] (http://www.deskgen.com) cloud platform. It brings together ten literature-based guide RNA scoring parameters and functions. It is unique to other design tools because it can simultaneously visualize all guides for a given gene according to four pre-computed guide RNA design parameters at a time. The tool can be used to support various experimental applications by accelerating and improving the guide design process. As new scores are published, the tool will be updated to accommodate, utilize and compare the latest algorithms.

## Abbreviations

CRISPR: Clustered regularly interspaced short palindromic repeats
DSB: Double-stranded break
Indel: Insertion/deletion mutation
MCDS: Master coding DNA sequence
NHEJ: Nonhomologous end joining
PAM: Protospacer adjacent motif
PPS: Percent peptide score
sgRNA: Single guide RNA
SpCas9: *Streptococcus pyogenes Cas9*
UI: User interface
