# Erratum to: Guide Picker is a comprehensive design tool for visualizing and selecting guides for CRISPR experiments

**DOI:** 10.1186/s12859-017-1618-8

**Published:** 2017-04-04

**Authors:** Soren H. Hough, Kris Kancleris, Leigh Brody, Neil Humphryes-Kirilov, Joseph Wolanski, Keith Dunaway, Ayokunmi Ajetunmobi, Victor Dillard

**Affiliations:** Desktop Genetics, Ltd., 28 Hanbury Street, London, E1 6QR UK

## Erratum

Following publication of this article [1], it has come to our attention that a caption for figure 6 is incorrect. Presently, it reads *“Fig. 6 Designing a Tiling Experiment with Guide Picker. a First, guides are plotted along the length of the MCDS using percent peptide on the x-axis. The y-axis is set to Doench 2016 Positionless. b Filtering guides to a strict score threshold (e.g.* ***<*** *55) offers a panel of guides that still target along the full length of the MCDS. c The right-hand plot can then be set and filtered according to other guide parameters, such as GC (30–70%) content and Hsu 2013 (* ***<*** *68) off-target scoring. d The guides in this range can then be highlighted to verify that this select group of guides still target along the full length of the MCDS in the left-hand plot”.* However, the less than (<) symbols should be more than (>) symbols and the caption should read, “*Fig. 6 Designing a Tiling Experiment with Guide Picker. a First, guides are plotted along the length of the MCDS using percent peptide on the x-axis. The y-axis is set to Doench 2016 Positionless. b Filtering guides to a strict score threshold (e.g.* ***>55*** *) offers a panel of guides that still target along the full length of the MCDS. c The right-hand plot can then be set and filtered according to other guide parameters, such as GC (30–70%) content and Hsu 2013 (* ***>68*** *) off-target scoring. d The guides in this range can then be highlighted to verify that this select group of guides still target along the full length of the MCDS in the left-hand plot.”*
